# The impact of maternal asthma on the preterm infants' gut metabolome and microbiome (MAP study)

**DOI:** 10.1038/s41598-022-10276-y

**Published:** 2022-04-19

**Authors:** Shiyu S. Bai-Tong, Megan S. Thoemmes, Kelly C. Weldon, Diba Motazavi, Jessica Kitsen, Shalisa Hansen, Annalee Furst, Bob Geng, Se Jin Song, Jack A. Gilbert, Lars Bode, Pieter C. Dorrestein, Rob Knight, Sydney A. Leibel, Sandra L. Leibel

**Affiliations:** 1 Division of Neonatology, Rady Children’s Hospital, University of California, San Diego, San Diego, CA USA; 2 Department of Pediatrics and Scripps Institution of Oceanography, University of California, San Diego, La Jolla, CA USA; 3 Center for Microbiome Innovation, University of California San Diego, La Jolla, CA USA; 4 Collaborative Mass Spectrometry Innovation Center, University of California, San Diego, La Jolla, CA USA; 5 Division of Allergy and Immunology, Rady Children’s Hospital, University of California, San Diego, San Diego, CA USA; 6 Division of Neonatology, Department of Pediatrics, University of California San Diego, La Jolla, CA USA; 7 Mother-Milk-Infant Center of Research Excellence (MOMI CORE), University of California, San Diego, La Jolla, CA USA; 8 Herbert Wertheim School of Public Health and Human Longevity Science, University of California, San Diego, La Jolla, CA USA

**Keywords:** Translational research, Microbiology, Medical research

## Abstract

Preterm infants are at a greater risk for the development of asthma and atopic disease, which can lead to lifelong negative health consequences. This may be due, in part, to alterations that occur in the gut microbiome and metabolome during their stay in the Neonatal Intensive Care Unit (NICU). To explore the differential roles of family history (i.e., predisposition due to maternal asthma diagnosis) and hospital-related environmental and clinical factors that alter microbial exposures early in life, we considered a unique cohort of preterm infants born ≤ 34 weeks gestational age from two local level III NICUs, as part of the MAP (Microbiome, Atopic disease, and Prematurity) Study. From MAP participants, we chose a sub-cohort of infants whose mothers had a history of asthma and matched gestational age and sex to infants of mothers without a history of asthma diagnosis (control). We performed a prospective, paired metagenomic and metabolomic analysis of stool and milk feed samples collected at birth, 2 weeks, and 6 weeks postnatal age. Although there were clinical factors associated with shifts in the diversity and composition of stool-associated bacterial communities, maternal asthma diagnosis did not play an observable role in shaping the infant gut microbiome during the study period. There were significant differences, however, in the metabolite profile between the maternal asthma and control groups at 6 weeks postnatal age. The most notable changes occurred in the linoleic acid spectral network, which plays a role in inflammatory and immune pathways, suggesting early metabolomic changes in the gut of preterm infants born to mothers with a history of asthma. Our pilot study suggests that a history of maternal asthma alters a preterm infants’ metabolomic pathways in the gut, as early as the first 6 weeks of life.

## Introduction

Asthma is an immune-mediated multifactorial disease that is influenced by both genetic and environmental factors. In particular, a maternal history of asthma is thought to increase the risk of asthma development in their children^1,2^. The composition of the gut microbiome, which is shaped by environmental exposure, is associated with the development of allergic diseases among children, likely due to its modulation of the innate and adaptive immune system^3–6^. For example, immature gut microbial composition at 1-year of age is associated with an increased risk of asthma by age five in the offspring of mothers with an asthma diagnosis^7^. Additionally, metabolites, which are reflective of genetic and environmental factors, play a critical role in disease pathogenesis and are implicated in common neonatal conditions and atopic diseases, such as asthma^8–10^. However, the mechanisms by which gut-associated metabolites influence lung health remains unclear^11^. Despite mounting evidence of the importance of genetic and environmental components in asthma pathogenesis, their relative effects are rarely studied together, and there have yet to be any studies that consider their combined importance in shaping both the microbiome and metabolome of the preterm infant gut over time. Therefore, the characterization of gut-associated bacteria and metabolites in response to these combined factors might allow us to evaluate childhood asthma risk more accurately in preterm infants.

The assembly of the microbiome in the infant gut represents a de novo microbial community influenced by various environmental factors^12,13^. In healthy term infants, these factors include breastfeeding, contact with adults, whether they have older siblings in the home, and pet ownership^14–17^. Preterm infants experience dramatically different exposures compared to healthy term infants. Prior to delivery, preterm fetuses are commonly exposed to antenatal antibiotics and steroids, and preterm infants are delivered via Caesarean section (C-section) more frequently than term infants^18^. Postnatally, infants admitted to the NICU are exposed to antibiotics, have decreased contact with parents and siblings, and experience delays in the introduction of enteral and oral feeds. These factors have been shown to alter the gut microbiome, resulting in higher numbers of *Enterobacteriaceae* and *Clostridiaceae*, and the absence of *Bifidobacteriaceae* and *Lactobacillaceae*, a pattern consistent with gut dysbiosis^14,19,20^. Alterations to the gut microbiome are of particular concern among preterm infants, because dysbiosis increases the risk of development of allergic diseases, such as asthma^20^.

The intestinal microbiota interacts with the immune system through the production of metabolites, which can be taken up by immune and epithelial cells^21,22^. For example, short chain fatty acids, produced by a variety of microbes during fermentation of dietary fiber, are protective against food allergy and pulmonary allergic inflammation in mice^23–26^, and it has also been shown that polyunsaturated fatty acids (PUFA) interact with human microbes in asthma pathogenesis^23^. Understanding the relevant metabolomic mechanisms in gut microbiome–host interactions and gut dysbiosis would allow for new insights into the pathogenesis of childhood asthma and the development of critical clinical applications.

To explore the impact of maternal asthma diagnosis on the gut microbiome and metabolome in preterm infants, we recruited preterm infants less than or equal to 34 weeks gestational age into the Microbiome, Atopic Disease, Prematurity (MAP) Study from two level III NICUs in San Diego County, California, USA. We examined a sub-cohort of infants born to mothers with a history of asthma and compared them to preterm infants with no maternal history of asthma diagnosis. We analyzed stool and milk samples from three time points: at birth, 2 weeks, and 4–6 weeks postnatal age, during their NICU stay. We then compared differences in bacterial community structure and metabolomic profiles between maternal asthma and control patients, as well as assessed a variety of clinical factors associated with their hospital care.

## Results

### MAP patient characteristics

**Figure 1:**
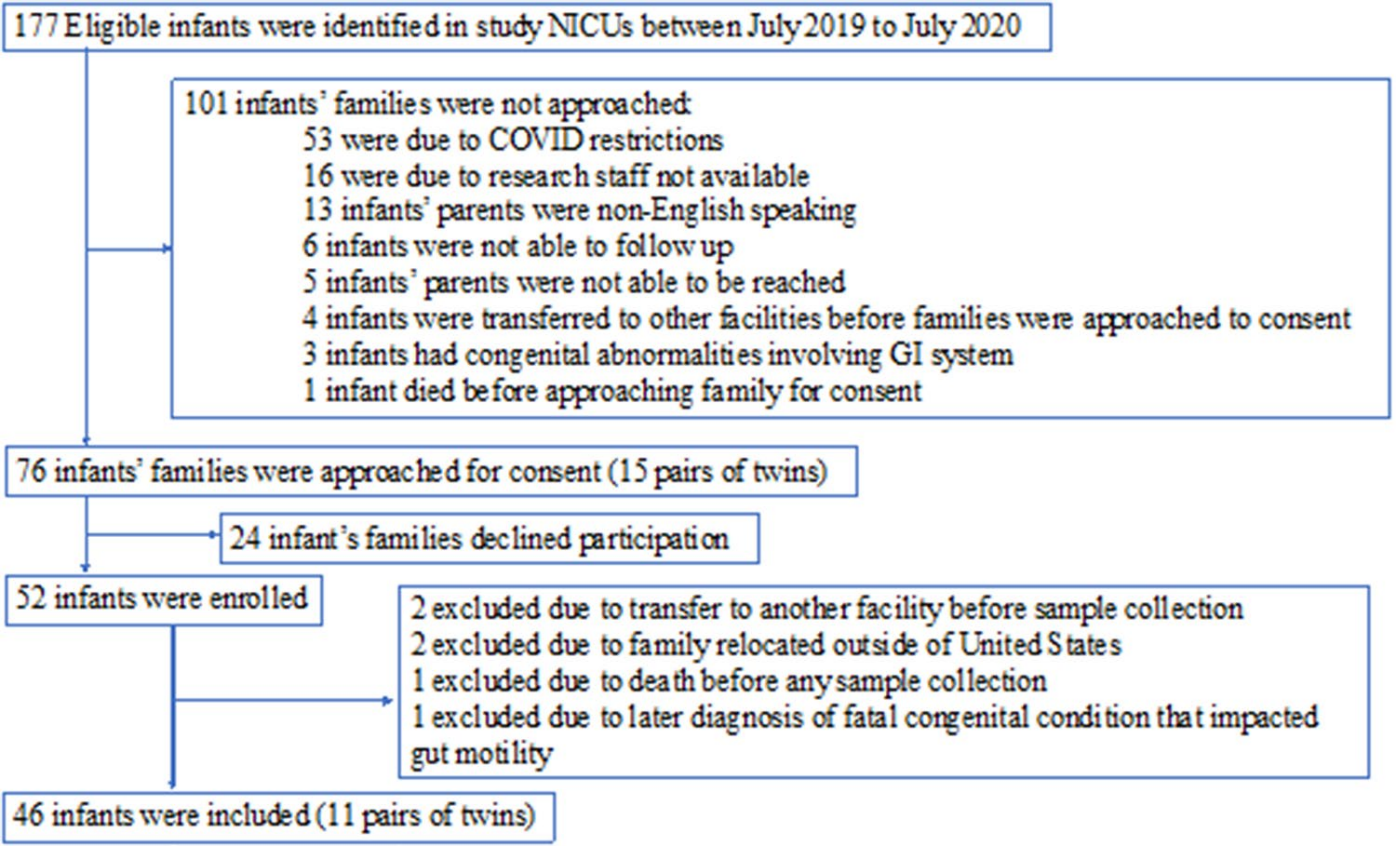
Outline of MAP study recruitment.

One hundred and seventy-seven eligible infants were identified in two level III NICUs, and 52 infants were recruited. Of these, six infants were excluded prior to sample collection, resulting in 46 infants completing the NICU portion of the MAP study (Fig. 1). All 35 mothers, including 11 with twin births, received antenatal betamethasone. Seventy-seven percent of mothers had a C-section. Thirty-one percent of mothers had preterm rupture of membranes and received latency antibiotics. Eleven percent received antibiotics for chorioamnionitis. Twenty-three percent of mothers received antibiotics earlier during pregnancy for other reasons. In addition, most mothers received antibiotics preoperatively for C-section or Group-B streptococcus (GBS) prophylaxis for vaginal delivery during the intrapartum period. Overall, only one mother did not receive any antibiotics prior to delivery (Supplementary Table 1).

The average gestational age for the 46 infants in the cohort was 31.0 weeks, ranging from 23.3 to 34.0 weeks. The average weight was 1591 g, ranging from 720 to 2290 g. Forty-three percent of the infants were female. Most of the infants were Caucasian (39%), followed by Hispanic (22%), and Asian (6%). Sixteen infants (35%) required intubation while 14 infants (30%) received surfactant. Thirty percent of infants required systemic antibiotics for more than 2 days. Forty-two (91%) infants experienced at least one episode of breastfeeding (nutritive or non-nutritive) during their NICU stay. Only 17 infants (40%) were discharged home primarily receiving maternal breast milk (Supplementary Table 2).

### Characteristics of maternal asthma and control sub-cohort

**Figure 2:**
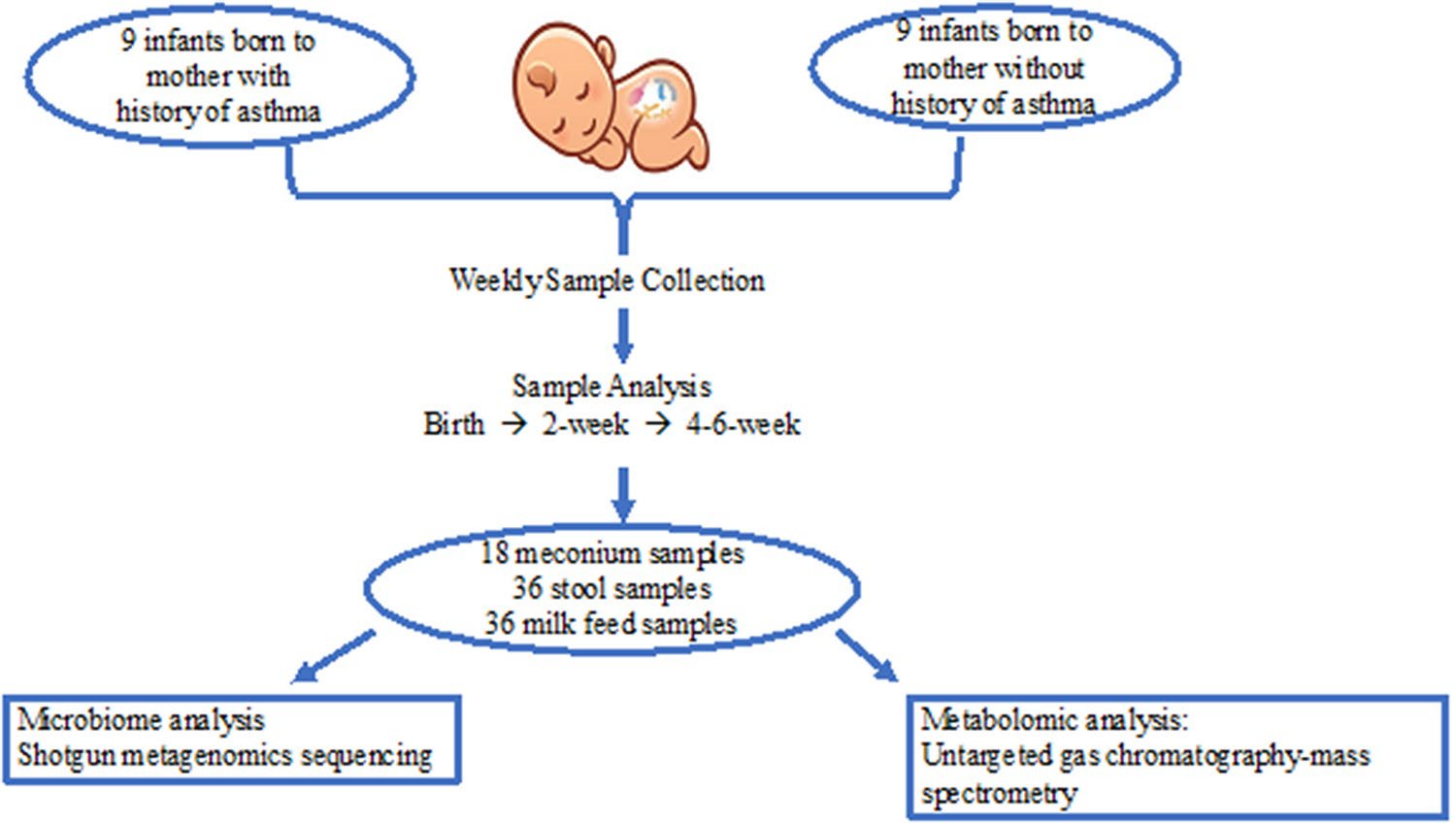
Maternal asthma pilot analysis study design.

For the pilot sub-cohort analysis, we identified nine preterm infants born to mothers with a self-reported history of asthma, as well as a gestational age matched control group without a maternal asthma diagnosis. The average birth weight in the control group was 1381 g compared to 1242 g in the maternal asthma group. All recorded patient characteristics were similar between the groups (*p* > 0.05), including the average gestational age, birthweight, APGAR scores, pairs of twins, rate of C-section, maternal antibiotic exposure, and infant antibiotic exposure (Table 1). All infants received maternal milk or donor human milk, human milk fortified by bovine-based fortifier, or preterm formula based on the neonatal units’ feeding policy, the infant’s clinical status, and the infant’s corrected gestational age at the time of sample collection. For each subject, three longitudinal stool samples collected from birth (timepoint 1), 2 weeks of age (timepoint 2), 4–6 weeks of age (timepoint 3), and two longitudinal milk feed samples from timepoints 2 and 3 were analyzed. A total of 54 stool samples, including 18 meconium samples, and 36 milk feed samples were characterized (Fig. 2).

**Table 1:** Clinical characteristics of the control and maternal asthma group.

Characteristics	Infant with maternal history of asthma n = 9	Infant without maternal history of asthma (control) n = 9
Gestational Age, average weeks (range)	29.7 (24.1–34)	29.6 (24.3–32.6)
Average weight, g (range)	1242 (575–2290)	1381 (605–2320)
Male infants, n	5	2
C-section, n	8	5
Prenatal Betamethasone, n	9	9
Premature Rupture of membrane, n	1	2
APGAR, average (range) at 1 MOL	5 (2–8)	6 (1–8)
APGAR average (range) at 5 MOL	8 (3–9)	7 (3–9)
Pair of Twins	2	1
Family ownership of dog, n	2	6
Older siblings in house, n	2	6
Current smoking in household, n	2	1
Maternal antibiotics during pregnancy, n	4	5
Maternal antibiotics during delivery, n	9	7
Infant received antibiotics during study period	7	4
Infant with required antibiotics for more than 2 days during study period	4	4
Intraventricular hemorrhage	3	2
ROP	1	0
Necrotizing enterocolitis	0	0
PDA required medical treatment	1	2
Needing home oxygen	1	0
Needing G-tube	1	3

*MOL* minute of life, *ROP* retinopathy of prematurity, *PDA* patent ductus arteriosus, *G-tube* gastrostomy tube.

### Stool-associated bacterial metagenome and metabolome

There were 2456 unique operational genomic units (OGUs; defined as sequence alignment hits to individual reference genomes) represented across all stool samples (ranging from 147 to 546 OGUs/sample). There was no observed association between maternal asthma diagnosis and bacterial richness or community composition in infant stool samples (*p* = 0.66, *p* = 0.398). When assessing changes in bacterial profiles at different timepoints, we did not observe any differentiation in communities between the maternal asthma and control groups, except at timepoint 2 (*p* = 0.0481). However, this difference was not observed at the third timepoint (*p* = 0.841), suggesting that there are no observable differences in the preterm infant gut microbiome after 6 weeks of stay in the NICU based on a history of maternal asthma diagnosis**.**

**Figure 3:**
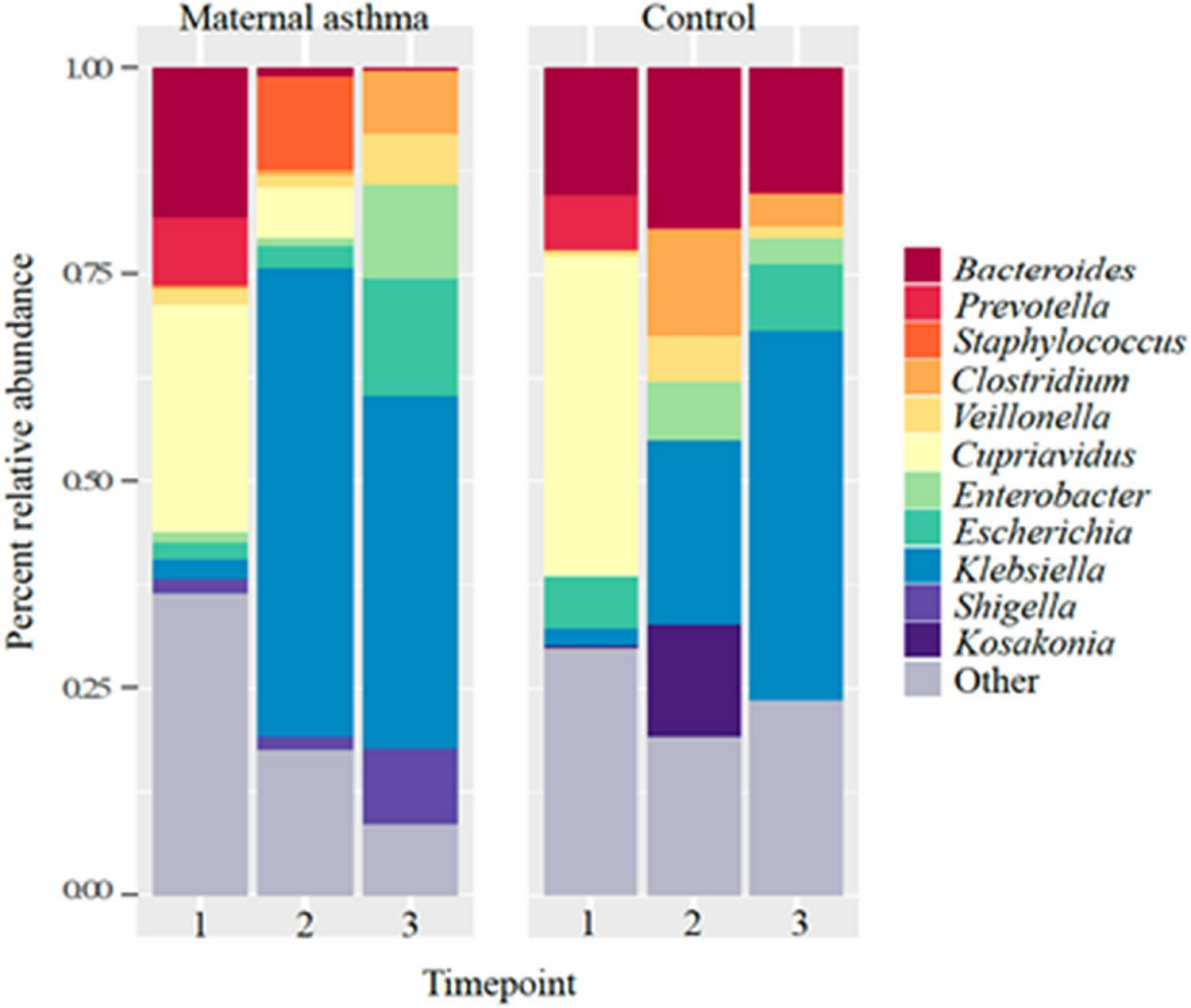
Stool microbiome longitudinal changes between maternal asthma and control group based on shotgun metagenomics.

Stool Bacteria in the genus *Klebsiella*, family *Enterobacteriaceae*, was highly abundant in stool samples from both the maternal asthma (35.4%) and control groups (22.0%) across all time points (Fig. 3). In the maternal asthma group, *Klebsiella* represented 2.3%, 56.6% and 42.5% of sequences at birth, 2 weeks, and 4–6 weeks postnatal age, respectively; similar to what was observed at the same timepoints in the control group (timepoints 1: 2.1%; *p* = 0.913, 2: 22.3%; *p* = 0.09, and 3: 44.5%; *p* = 0.918). The relative abundance of *Bacteroides* sharply decreased in the maternal asthma group over time (timepoints 1: 18.1%, 2: 0.9%, and 3: 0.3%), where this taxon stayed relatively stable and abundant in the control group (timepoints 1: 15.4%, 2: 19.2%, and 3: 15.1%; Fig. 3). Yet, though there were notable differences in the abundance of *Bacteroides* between the maternal asthma and control groups at timepoints 2 and 3, these differences were not significant (timepoints 2: *p* = 0.06 and 3: *p* = 0.187), potentially due to an uneven distribution of *Bacteroides* across samples and/or small sample size. *Bacteroides fragilis*, whose early intestinal colonization in term infants is associated with increased risk of asthma^27^, comprised < 0.5% of all sequences. Lastly, maternal asthma and control groups had very low abundances of *Bifidobacteriaceae* and *Lactobacillaceae*, at less than 1% of reads in stool samples from both groups. All taxa abundance comparisons were calculated with Kruskal–Wallis tests.

When considering additional clinical factors, bacterial richness and community composition were significantly correlated with timepoint (richness: *p* = 0.002, community composition: *p* = 0.001) and infant antibiotic exposure (richness: *p* = 0.009, community composition: *p* = 0.011). In addition, there was a strong differentiation in bacterial community composition after the initiation of bottle (*p* = 0.014) and breast feeding (*p* = 0.014), indicating changes to the stool microbiome at the onset of oral feeding, following full enteral feeds via a nasogastric tube. However, these differences were not apparent by timepoint 3, in which there were no observed differences in bacterial community structure due to infant bottle feeding (*p* = 0.34) or breast feeding (*p* = 0.52). All richness comparisons were calculated with Kruskal–Wallis, and community composition comparisons were done with a permutational multivariate analysis of variance (PERMANOVA).

**Figure 4:**
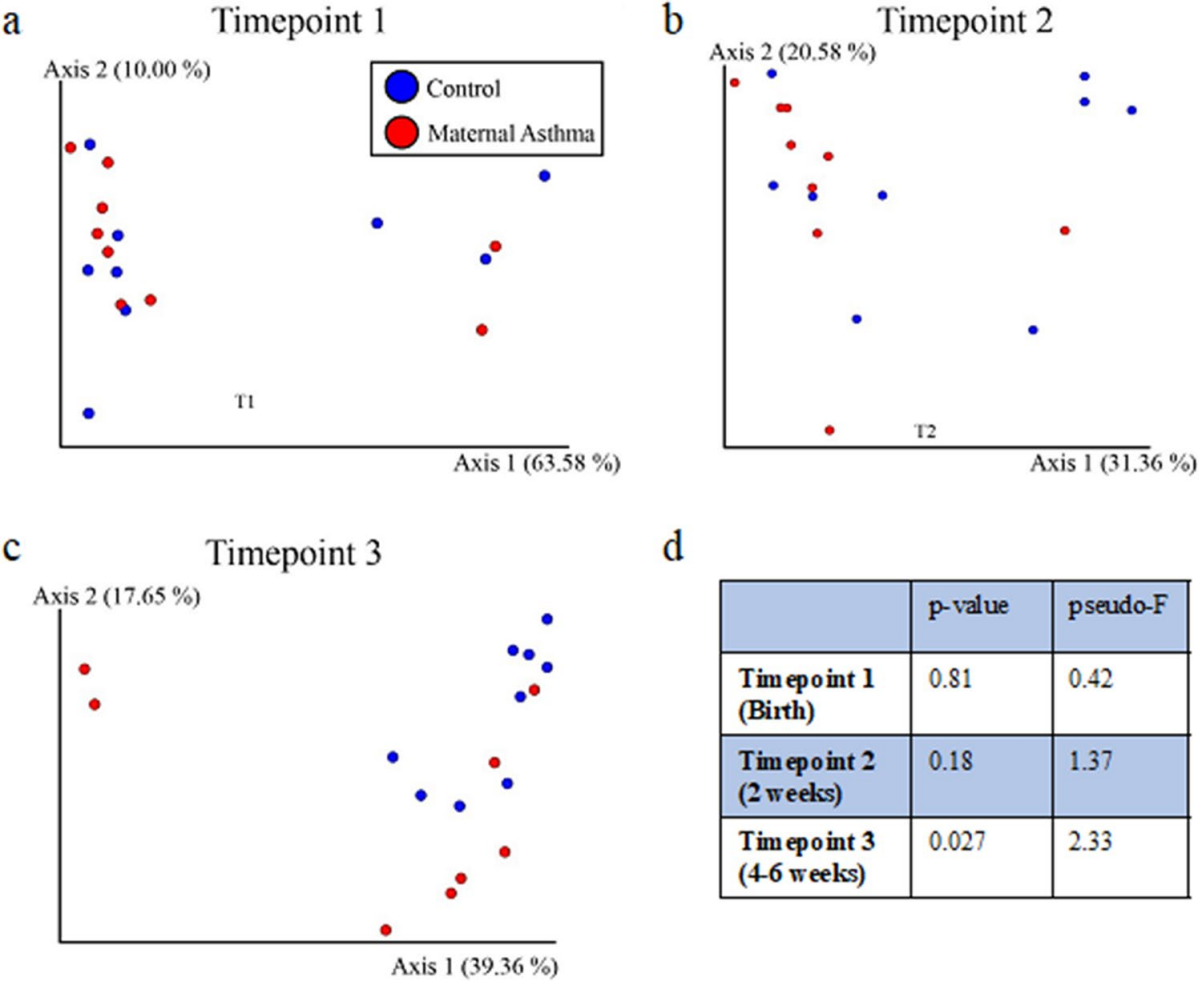
Stool metabolomic profile between maternal asthma and control group. Metabolomic profiles differentiated over time and became statistically significant at the third timepoint. (**a**–**c**) consist of stool data PcoA plots using a Bray Curtis distance metric on each timepoint. (**d**) Contains the PERMANOVA *p* values and pseudo-F statistics between maternal asthma samples and control samples for each timepoint, calculated using the Bray Curtis distance metric.

**Figure 5:**
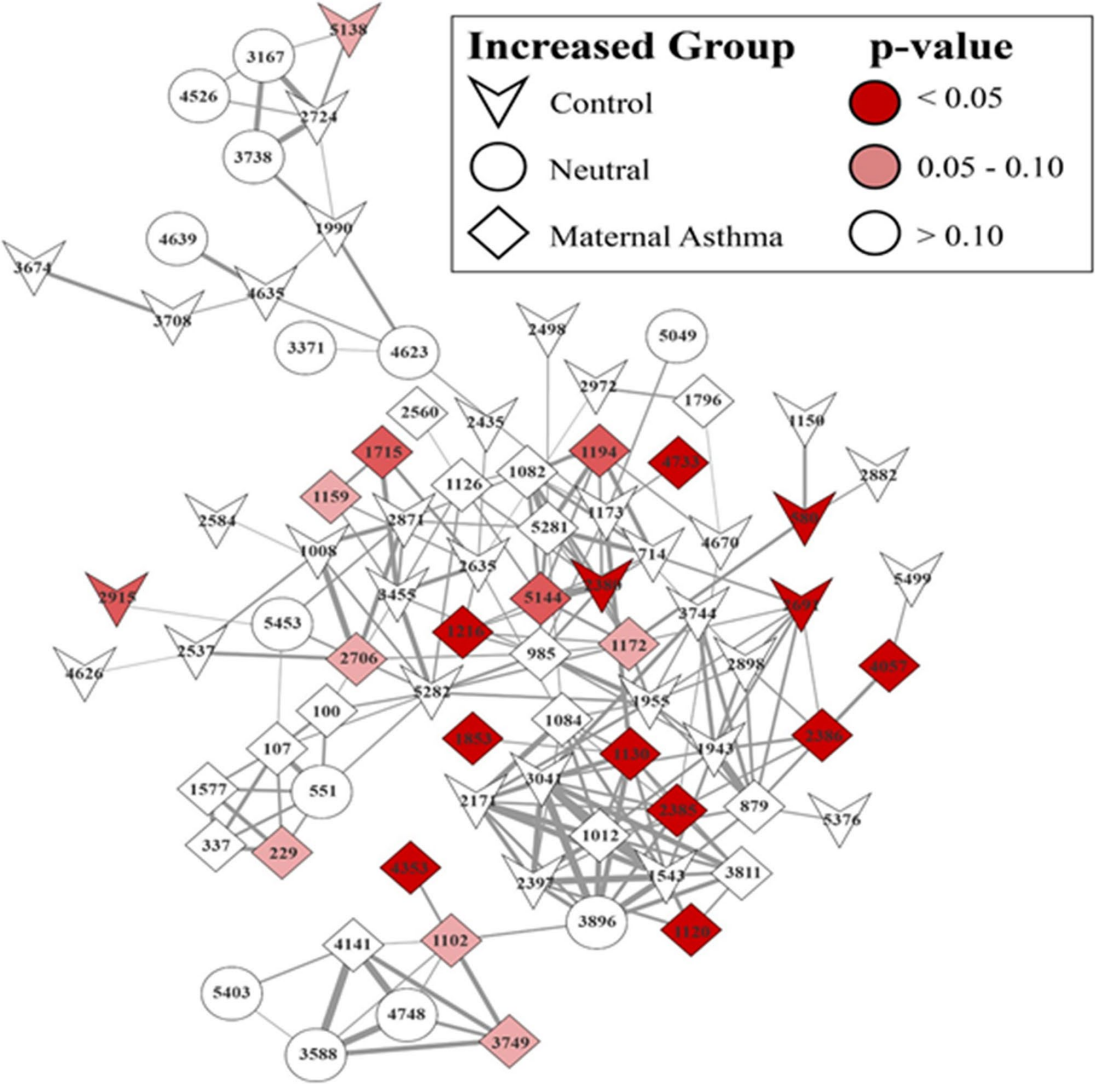
Stool linoleic acid network at timepoint 3 (4–6 weeks postnatal age). Multiple chemicals in the linoleic acid network differ significantly between the maternal asthma group and control group. The shape indicates which group the compound is increased based off median values. If the compound is indicated as neutral, both groups median values were at zero. The coloring indicates if this increase is significant based on a Dunn’s Test (multiple test corrected Kruskal Wallis). The widths of the lines connecting the compounds are determined by the spectral similarity cosine score, with the widest line being a score of 1. Compound annotation (from a GNPS library search) and additional information can be found in Supplementary Table 3.

Metabolomic analysis of the stool samples showed significant differences between the maternal asthma group and the control group at 4–6 weeks postnatal age (PERMANOVA; timepoint 3: *p* = 0.027) but not at earlier timepoints (*p* = 0.81 at timepoint 1, *p* = 0.18 at timepoint 2) (Fig. 4a–d). The stool samples were then classified between the maternal asthma group and the control group using a random forest sample classification analysis. The supervised machine learning sample classifier was able to identify multiple metabolites in the linoleic acid network that were of importance in the testing and training of the dataset. This model resulted in an out of bag error of 0.3333. A molecular network showed the compounds of importance, with many differences between the maternal asthma group and the control group at the 4–6-week timepoint (Fig. 5). In addition, stool-associated metabolites differed based on infant antibiotic use (*p* = 0.001, pseudo-F = 5.27), in agreement with the metagenomic data, but they did not differ after initiation of bottle or breastfeeding (*p* = 0.07; *p* = 0.31). In the primary untargeted metabolomic analysis, lacto-n-fucopentaose (LNFP) isomer, a human milk oligosaccharide (HMO), was initially implicated in the machine learning differentiation between the maternal asthma group and the control group, but further analysis with a Kruskal–Wallis test did not show a statistical significance in LNFP isomer level in stool samples between the two groups at timepoint 3 (*p* = 0.12).

### Milk feed-associated bacterial metagenome, metabolome and human milk oligosaccharides

**Figure 6:**
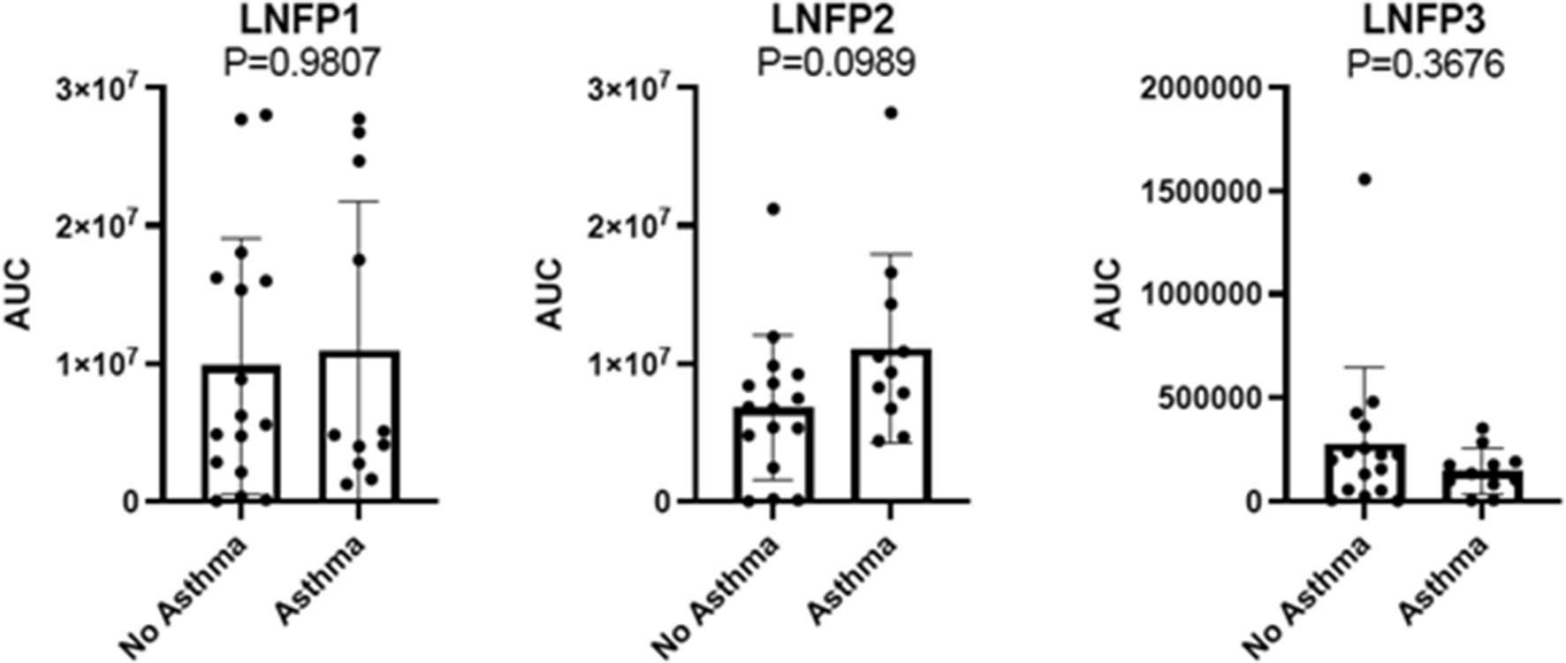
HMO analysis of milk feed samples. No significant differences were detected in the HMO concentrations between the groups. Significance was calculated using the Mann–Whitney test.

Milk feed samples were analyzed at timepoints 2 and 3 only, as most preterm infants did not receive enteral feed immediately after birth (timepoint 1). Milk feed bacterial compositions were similar between the maternal asthma and control groups (timepoint 2: *p* = 1.0, timepoint 3: *p* = 0.31, Table 2, Supplementary Table 4). Among milk samples, OGU abundance ranged from144 to 439 unique taxa per sample. Bacterial richness, bacterial community composition, and metabolite profiles were not correlated with any of the clinical variables tested in our study, including between maternal asthma and control groups (richness: *p* = 0.663, bacterial community composition: *p* = 0.369). However, since there was initially evidence of differences between the maternal asthma and control groups for the HMO LNFP isomers in infant stool, and this HMO plays a role in supporting gut microbiome health, we examined the composition of the HMOs in the milk feed samples. HMO analysis from the two groups (n = 16 for control group and n = 11 for maternal asthma group) showed no differences in LNFP 3 levels (Mann–Whitney: *p* = 0.37), but there was a trend showing less LNFP 3 in the maternal asthma group (Fig. 6).

**Table 2:** Milk feed composition of the two groups at both timepoints 2 and 3 (*p* = 0.31).

Milk feed composition	Maternal asthma, n = 18	Control, n = 18
Maternal milk	1	3
Fortified maternal milk	11	9
Fortified donor milk	2	2
Fortified mixed maternal and donor milk	1	0
Preterm formula	1	4
Unknown	2	0

Significance was assessed using Chi-square test.

## Discussion

Here, we present a unique paired dataset of bacterial communities and metabolites from stool and milk feed samples collected from preterm infants that are at risk for asthma development. We demonstrated that preterm infants born to mothers with a history of asthma showed different stool metabolomic profiles compared to those without a maternal asthma diagnosis, particularly at 4–6 weeks postnatal age. We did not observe a similar longitudinal change in the stool bacterial communities; however, this could be due to small sample sizes and a short period of collection. For milk feed samples, we did not observe differences in the bacterial, metabolomic, or HMO profiles between the maternal asthma and control groups.

The overall microbial profile from our stool samples were consistent with what has been reported in previous studies. *Klebsiella* was the most abundant genus observed among stool samples, while genera from families *Bifidobacteriaceae* and *Lactobacillaceae* were undetected or minimal, a pattern commonly observed in premature infants^19,20,28^. In addition, stool microbial patterns showed significant change over time^14,19^, and differences in bacterial community richness and composition, as well as the metabolome, were associated with an infant’s exposure to antibiotics at all timepoints^29–31^.

In comparing the stool-associated bacteria between maternal asthma and control groups, we observed a loss of *Bacteroides* in the maternal asthma group over time, while the abundance of *Bacteroides* across samples in the control group remained stable. This is particularly notable as the abundance of *Bacteroides* bacteria in the gut provides a protective effect in the development of immune disease^32–35^. Where we do acknowledge the small sample size of our sub-cohort could limit our interpretation of this data and that the differences between groups were not statistically significant, the observed pattern suggests that gut dysbiosis could begin as early as the neonatal period in populations at risk of atopic disease. Additionally, though C-section births have previously been shown to impact the neonatal gut microbiome, including a decrease in the colonization of *Bacteroidetes* among infants^36,37^. we did not observe any evidence that these differences were due to mode of delivery. The rate of C-section was not statistically different between the maternal asthma and control groups, and the samples with the highest abundance of *Bacteroides* (at the first timepoint) were both from individuals that were born by C-section.

There is growing evidence that microbiome-metabolome interactions are involved in the immune system ontogeny and are important in asthma development^23^. Although maternal asthma history is known to increase an offspring’s risk for developing childhood asthma, the mechanism is unclear and multifactorial^1,2^. However, our results might provide insight into asthma disease pathogenesis, in which metabolites in the linoleic acid family differed in the stool samples between preterm infants born to mothers with a history of asthma compared to those without. Linoleic acid (18:2w6; cis, cis-9,12-octadecadienoic acid) is a diet derived polyunsaturated fatty acid (PUFA) whose derivatives are involved in cell signaling^38^. As the parent compound for the family of omega-6 PUFA, linoleic acid can give rise to inflammatory eicosanoids^23,38^. Linoleic acid metabolites have been implicated in steroid-resistant asthma, by causing steroid unresponsiveness in a mouse model of allergic airway inflammation^39^. Additionally, metabolomic analysis of serum from adult patients with asthma identified linoleic acid as being more abundant in severe asthma patients compared to healthy controls^10^. Similarly, infants with bronchiolitis who had greater abundances of linoleic acid in their nasopharyngeal samples were more likely to develop wheezing and asthma later in life^40^. Although microbes do not produce PUFAs, there is increasing evidence of PUFA interactions with human gut microbes in asthma pathogenesis^23^. One study showed that supplementation of the probiotic *Lactobacillus rhamnosus* GG in infants increased the level of stool omega-3 fatty acid, which is an anti-inflammatory PUFA^41^. Our study found higher linoleic acid metabolites in the stool samples from preterm infants in the maternal asthma group, suggesting a pro-inflammatory process starting as early as the neonatal period among this at-risk population. Further, we found that the maternal asthma group had lower levels of LNFP 3 in the milk feeds; however, they did not reach statistical significance. Lacto-n-fucopentaose (LNFP) isomers are human milk oligosaccharides (HMO) that exert immunological effects as prebiotic substrates for the gut microbiota^42,43^. High LNFP 3 levels in maternal breast milk are protective against the development of cow’s milk protein allergy in infants^44^.

The MAP study cohort encapsulated robust maternal and infant clinical information, corresponding to the microbial and metabolomic analyses of the stool and milk feeds. In addition, our sub-cohort selected for maternal asthma analysis was a single-batch paired analysis of the microbiome and metabolome in stool and milk feed samples, with an additional HMO analysis of milk feed samples. This approach minimized batch effects and allowed for direct comparison and correlation of clinical, microbiome, metabolome, and HMO data. However, there were several limitations to our study. First, although there were 54 stool samples and 36 milk samples analyzed longitudinally, the sample size of our pilot sub-cohort maternal asthma groups was small with only nine patients in each group. We were restricted by the number of mothers with a history of asthma, with concomitant limits on statistical power which might overlook the impact of clinical factors from metadata on the microbiome, as well as metabolic patterns observed between the two groups. Future studies will aim to specifically recruit more mothers with a history of asthma to increase the statistical power and further distinguish the gut microbial and metabolomic patterns in preterm infants at risk for atopy. Additionally, the microbial and metabolomic findings in our sub-cohort could be an NICU specific phenomenon, so follow-up outpatient data will be important to consider when providing context to the clinical significance of our findings. In addition, because a preterm infant’s gut microbiota is characterized by delayed colonization of microbes and low biodiversity^45,46^, our small sample size and limited duration of sample collection could contribute to the lack of distinction in stool microbial profiles between two groups. This small sample size also limits the significance of our linoleic acid findings as low metabolite abundances could lead to the significant *p* values found in Fig. 5 and Supplementary Table 3. The untargeted metabolomics design was optimized to identify small metabolites in positive ionization mode, limiting the current methods ability to quantify polyunsaturated fatty acids. Our analysis did identify differences in the linoleic acid network, but further analysis would require more optimized mass spectrometry methods to gain a better quantification of these compounds. Additionally, a limitation in the HMO analysis was that milk feed samples were fortified with bovine-based fortifiers. Although this analysis accurately reflects the standard clinical practice of how preterm babies are fed in the study NICU, the bovine fortifier contains maltodextrin, a polysaccharide that is labelled together with HMOs and interferes with the quantification of absolute concentrations of HMOs. Although it would have been ideal to analyze unfortified milk feeds at the various timepoints, this would not have accurately replicated the interactions of fortified milk feeds with the gut microbiome in the preterm infant in a real world NICU. To improve HMO quantification, future studies should include milk sampling prior to the addition of bovine fortifiers.

In conclusion, our study demonstrated novel differences in stool metabolites between preterm infants born to mothers with and without asthma. These differences were observed longitudinally from birth to 4–6 weeks postnatal age, with those differences becoming more apparent over time. The linoleic acid metabolic network is known to play a role in the development of asthma during the critical window of immune development. Follow-up data from the MAP cohort in these same infants after discharge will longitudinally assess the allergic sensitization patterns at one year of age and development of asthma by 4–6 years of age to better understand the clinical implication of our results. Thus, with this study, we have laid the groundwork to potentially identify preterm infants in the NICU at risk for allergic sensitization and the development of asthma. This could lead to the development of therapeutic interventions during a critical window of immune development to reduce the risk of childhood asthma in this susceptible population.

## Methods

### Ethics statement

The University of California San Diego Institutional Review Board approved all study protocols (IRB approval #181711). All research, experiments and data analysis were performed in accordance with the University of California guidelines and regulations. Participant parents/guardians provided written, informed consent prior to enrollment in this research project. Furthermore, the study “The Association Between Milk Feedings in the Preterm Population, the Microbiome and Risk of Atopic Disease, MAP (Microbiome, Atopic Disease, Prematurity) Study” was registered in the ClinicalTrials.gov database (NCT04835935).

### MAP study recruitment, sample collection, outcome measures, and statistical analysis

We performed a prospective observational study at two level III NICUs in San Diego, California, USA from June 2019 to September 2020. Preterm infants born at 34 weeks gestational age or less were eligible for inclusion in this study. Infants were excluded if they were born with congenital anomalies that impacted the gastrointestinal system or those unable to follow up (i.e., infants born via surrogacy, infants’ family reside in different cities). The MAP study sample size estimation was based on effect size for beta and alpha diversity differences, as previously described^41^. Utilizing effect size for beta diversity in Durack et al., we used a two-sample t-test, which implied an n = 16 would give a power of 0.8. Utilizing effect size for alpha diversity slope (0.14 vs. 0.22 over time between treatment groups), we calculated a one-way K means ANOVA which implied n = 8 in each group would give a power of 0.8. Considering that 30–50% of patients might be lost throughout the duration of our pilot study, we recruited a convenience sample size of 50 infants.

Samples were collected weekly from stool and milk feeds. Stool samples were swabbed using BD Falcon™ SWUBE™ Dual Swab (cotton tip) from soiled diapers and meconium was prioritized in the first week of life, where possible. Milk feed samples were taken from the feeding syringe or bottle before or after feeding events. Milk feeds were recorded based on which milk type the preterm infant was receiving at the time of sample collection and included unfortified human milk, fortified human milk, and preterm formulas. All collected samples were stored immediately after collection at 4 °C and transferred to − 80 °C within 12–36 h. Additionally, maternal demographic information was collected, including maternal dietary history during pregnancy, family history of atopic disease, and antenatal courses. Infant demographics collected included birth history and clinical course data (e.g., preterm morbidities, feeding history, and antibiotic usage).

Statistical analysis for clinical data was performed using GraphPad Prism 7 (Graphpad Software, Inc.). An unpaired Student-T test was used to analyze continuous clinical variables with a normal distribution. Either a Chi-square test or a Fisher’s exact test was used to compare categorical variables, where appropriate. Mann–Whitney U-test was employed to analyze continuous variables with non-normally distributed histograms.

### Maternal asthma sub-cohort

For the pilot sub-cohort, we identified nine preterm infants born to mothers with a self-reported history of asthma (present or past), as well as a control group without maternal asthma diagnosis, of which paired infants were of the similar gestational age. All 18 infants were born at the University of California, San Diego, Jacobs Medical Center. Meconium samples from birth and stool and milk samples from 2 and 4–6 weeks were analyzed for microbiome and metabolomic profiles. These time courses were chosen to include infants on minimal enteral feeds, full enteral feeds, and feeds taken orally. A total of 54 stool samples, including 18 meconium samples, and 36 milk feed samples were included in our analyses.

### Metagenomics

DNA was extracted from samples using the Qiagen MagAttract PowerSoil DNA kit, following the Earth Microbiome Project protocol^47^. Input DNA was quantified using a PicoGreen fluorescence assay (ThermoFisher, Inc) and library preparation was subsequently performed using a 1:10 miniaturized KapaHyperPlus protocol as previously described^48^. All samples were then normalized, based on a PicoGreen fluorescence assay, prior to sequencing on the Illumina NovaSeq platform (SP 300, PE150) at the Institute for Genomic Medicine (IGM), UC San Diego.

Raw metagenomic sequences were quality filtered and trimmed using fastp^49^, and human reads were filtered using minimap2^50^. Resulting reads were aligned using Bowtie2 and classified with the Woltka pipeline^51^ (Web of Life Toolkit App; v 0.1.1; https://github.com/qiyunzhu/woltka) against the Web of Life database^52^, resulting in a table of Operational Genomic Units (OGUs). Taxonomic profiles were analyzed in an R environment with the mctoolsr package (Leff, J. W. 2016. *mctoolsr: microbial community data analysis tools.* R package version 0.1.1.1. https://github.com/leffj/mctoolsr). Stool samples were rarefied to 14,000 reads, and breast milk samples were rarefied to 7400 reads for all downstream analyses. OGU richness was compared with Kruskal–Wallis. Additionally, differences in bacterial community composition were calculated among samples with Bray–Curtis dissimilarity^53^, weighted by OGU abundance and compared with a permutational multivariate analysis of variance (PERMANOVA), in which stool and milk samples were compared separately.

### Metabolomics

For untargeted metabolomics by LC–MS, metabolomics extraction and data acquisition, using LC–MS was completed in the Collaborative Mass Spectrometry Center at UC San Diego.

Stool sample swabs were transferred to a 96-deepwell plate over ice, and metabolites were extracted with a solution of 1:9 LCMS grade water to ethanol. Swabs were incubated in the solvent overnight at − 20 °C and removed from the wells after the incubation period. Breast milk samples were thawed and 50 µL was transferred to a new eppendorf 2 mL tube over ice. Milk-associated metabolites were extracted by adding 100% LCMS grade methanol to each sample to create a 1:4 sample to methanol volume for extraction.

The extracted metabolites were concentrated down and resuspended in a 1:1 methanol to water (LCMS grade) solution. An ultrahigh performance liquid chromatography system (Thermo Dionex Ultimate 3000 UHPLC) coupled to an ultrahigh resolution quadrupole time of flight (qToF) mass spectrometer (Bruker Daltonics MaXis HD) was used for data acquisition. Metabolomics data processing and feature detection was completed using MZmine software (http://mzmine.github.io/). Mzmine parameters were used as follows: Mass detection (MS1 noise level 1000, MS2 noise level 100); ADAP Chromatogram builder (Min group size 3, group intensity threshold 3000, min highest intensity 1000, m/z tolerance 0.01 m/z or 10 ppm); Chromatogram deconvolution using local minimum search (chromatographic threshold 0.01%, min in RT range 0.04 min, minimum relative height 0.01%, minimum absolute height 3000, min ratio of peak top/edge 2, peak duration range 0.05–0.50 min, m/z center calculation AUTO, m/z range for MS2 pairing 0.01 Da, RT range for MS2 pairing 0.1 min); Isotopic peak grouper (m/z tolerance 0.01 m/z, maximum charge 4, RT tolerance 0.3 min, representative isotope most intense), join aligner (m/z tolerance 0.01 m/z or 10 ppm, weight for m/z 75, RT tolerance 0.3 min, weight for RT 25); Gapfilling (intensity tolerance 20%, m/z tolerance 0.005 m/z or 10 ppm, RT tolerance 0.2 min), peak filter (area > 10,000). The resulting tables consisted of 5,536 metabolomics features. Library annotation and molecular networking was completed on the Global Natural Products Social Molecular Networking (GNPS) platform (https://gnps.ucsd.edu/ProteoSAFe/static/gnps-splash.jsp) feature based molecular networking workflow^54,55^. From the 5,536 features, 175 were annotated using the GNPS public libraries. The resulting tables were TIC normalized. Data visualizations and machine learning analyses (sample classification using a supervised learning technique which splits data into training and testing sets, then trains and tests the estimator using a stratified k-fold cross-validation scheme, then tests for classification accuracy) were completed using QIIME 2^56^ (https://qiime2.org/), and the Dunn’s Test was run on specific metabolites that were identified to be significantly different between the maternal asthma and control groups using R scripts.

### Human milk oligosaccharides

Concentrations of HMOs (mg/mL) were measured, as previously described^57^. Briefly, 20 µL of human milk were dried in a 96-well plate and oligosaccharides were fluorescently labeled with 2-aminobenzamide (2AB, Sigma) in a thermocycler heat block at 65 °C for exactly 2 h. The reaction was stopped abruptly by reducing the thermocycler temperature to 4 °C. The amount of 2AB was titrated to be in excess to account for the high and variable amount of lactose and other glycans in milk samples. Labeled oligosaccharides were analyzed by HPLC (Dionex Ultimate 3000, Dionex, now Thermo) on an amide-80 column (15 cm length, 2 mm inner diameter, 3 μm particle size, Tosoh Bioscience)) with a 50-mmol/L ammonium formate–acetonitrile buffer system. Separation was performed at 25 °C and monitored with a fluorescence detector at 360 nm excitation and 425 nm emission. Peak annotation was based on standard retention times of commercially available HMO standards and a synthetic HMO library and offline mass spectrometric analysis on a Thermo LCQ Duo Ion trap mass spectrometer equipped with a Nano-ESI-source. Concentrations were estimated by calculating area under the curve for each annotated HMO. Absolute quantification was not calculated because of fortifier interference. HMO concentrations between the groups were compared by Mann–Whitney test.

## Supplementary Information

Supplementary Information.

## Data Availability

Metagenomic raw sequence files and metadata tables are publicly available in the European Nucleotide Archive (ENA; Accession ID ERP136831), as well as in the Qiita repository (Study ID 13241). Additionally, all R code used for metagenomic analyses are available on GitHub at https://github.com/hillms/MAP. Metabolomic raw data, mzXML files spectral files (.mgf) and feature quantification tables can be found on the MassIVE (https://massive.ucsd.edu/) database, under the accession number MSV000086246. The feature based molecular networking job can be found here: https://gnps.ucsd.edu/ProteoSAFe/status.jsp?task=8a62828118e84fc39c30439425628627.
