# Supplementary material for: The impact of maternal asthma on the preterm infants' gut metabolome and microbiome (MAP study)
Source: Sci Rep. 2022 Apr 19;12:6437. doi: 10.1038/s41598-022-10276-y (PMC9018729; doi:10.1038/s41598-022-10276-y)
Supplement: Supplementary file 1 — Supplementary Information. [file 41598_2022_10276_MOESM1_ESM.pdf]

Supplementary Tables

The Impact of Maternal Asthma on the Preterm Infants' Gut Metabolome and Microbiome (MAP study)

Shiyu S. Bai-Tong[1], Megan S. Thoemmes[2], Kelly C. Weldon[3,4,] Diba Motazavi[5], Jessica Kitsen[5], Shalisa Hansen[3], Annalee Furst[6], Bob Geng[5], Se Jin Song[3], Jack A. Gilbert[2,3], Lars Bode[6,7], Pieter C. Dorrestein[3,4], Rob Knight[3,4], Sydney A. Leibel[5,8*], Sandra L. Leibel[1*]

1 Division of Neonatology, University of California, San Diego, Rady Children's Hospital, San Diego, CA, USA

2 Department of Pediatrics and Scripps Institution of Oceanography, University of California, San Diego, La Jolla, CA, USA

3 Center for Microbiome Innovation, University of California San Diego

4 Collaborative Mass Spectrometry Innovation Center, University of California, San Diego, La Jolla, CA, USA

5 Division of Allergy and Immunology, University of California, San Diego, Rady Children's Hospital, San Diego, CA, USA

6 Department of Pediatrics, Division of Neonatology, University of California San Diego,

7 Mother-Milk-Infant Center of Research Excellence (MOMI CORE), University of California, San Diego

8 Herbert Wertheim School of Public Health and Human Longevity Science, University of California, San Diego

Corresponding Authors:

Sandra L. Leibel, saleibel@health.ucsd.edu

Sydney A. Leibel, sleibel@health.ucsd.edu

Supplementary Table 1: Maternal History and Antenatal Course

N = 35 (11 pairs of twins)		
Average Age, year (range)	31	(20-46)
Betamethasone use, n (percentage)	35	(100%)
C-section, n (percentage)	27	(77%)
PPROM, n (percentage)	11	(31%)
Latency Antibiotic Use, n (percentage)	11	(31%)
Maternal Chorioamnionitis, n (percentage)	4	(11%)
Antibiotic Chorioamnionitis, n (percentage)	4	(11%)
Other antibiotic use during pregnancy,	8	(23%)
Pregnancy Complications, n (percentage)		
Diabetes	11	(31%)
Chronic hypertension	7	(20%)
Pre-eclampsia or HELLP	7	(20%)
Hypothyroidism	3	(9%)

Abbreviations: PPROM: premature prolonged rupture of membrane; HELLP: hemolysis, elevated liver enzymes, low platelet count.

Supplementary Table 2: Infant Demographics, Birth History, and NICU Course

N = 46		
Average Gestational Age, week (range)	31.0	(23.3 – 34.0)
Average Weight, g (range)	1591	(720 – 2290)
Male, n (percentage)	20	(43%)
Ethnicity, n (percentage):		
Caucasian	18	(39%)
African American	2	(4%)
Hispanic	10	(22%)
Asian	6	(13%)
Hawaiian or Pacific Islander	2	(4%)
Mixed Race	4	(8%)
Other	4	(8%)
Average Birth Length, cm (range)	40.3	(30.5 – 48.0)
Average Head Circumference, cm (range)	28.5	(18.0 – 33.5)
Median APGAR at 1 min, (range)	7	(1 – 9)
Median APGAR at 5 min, (range)	8	(3 – 9)
Median APGAR at 10 min, (range), N = 9	7	(5 – 8)
Number of Infants who required intubation, n (percentage)	16	(35%)
Average Intubation Duration, days (range)	4	(1 – 24)
Surfactant administration, n (percentage)	14	(30%)
Number of infants required systemic steroid, n (percentage)	2	(4%)
Number of infants required systemic antibiotics > 2 days, n (percentage)	14	(30%)
Number of infants required systemic antifungal, n (percentage)	4	(9%)
Number of infants required antacid, n (percentage)	1	(2%)
Date of Life when oral feeding was started, days (range)	22	(0-98)
Date of Life when non-nutritive breast feeding was started (N = 42), days (range)	23	(2 - 85)
Diet at Discharge (N = 43)		
Anti-reflux formula, n (percentage)	7	(16%)
Preterm formula, n (percentage)	5	(12%)
Preterm formula with minimal breast milk, n (percentage)	4	(9%)
Preterm formula and maternal breast milk, n (percentage)	10	(23%)
Maternal breast milk (supplemented with formula for increased calories), n (percentage)	17	(40%)
PDA requires treatment, n	4	
ROP, n	2	
IVH:		
Grade 1 and Grade 2, n	8	
Grade 3 and Grade 4, n	0	
Necrotizing enterocolitis, n	0	
SIP, n	0	
Home oxygen, n	1	
G-tube, n	4	

Abbreviations: ROP: retinopathy of prematurity, PDA: patent ductus arteriosus, G-tube: gastrostomy tube.

Supplementary Table 3: Linoleic acid network compound annotation, labels from Figure 5. Compound annotations from GNPS library search feature based molecular networking workflow. The m/z and retention time values were determined using feature dectection in MZmine (see methods for parameters). P-values calculated using a multiple test corrected Kruskal Wallis (Dunn's Test). The group with the highest median value is listed, with some features not showing a higher median value group as both medians were at zero.

SupTable_LinoleicAcid_Network

Label	Compound Annotation	m/z	Retenton Time (min)	p-value (Maternal Asthma vs Control)	Highest Median Value at T3
100	Jasmonic acid	193.1220	5.18	0.335923813	Maternal Asthma
107	Not Annotated	229.1434	5.22	0.563702862	Maternal Asthma
229	Not Annotated	286.1652	4.67	0.083264517	Maternal Asthma
337	Not Annotated	300.1806	4.94	0.12365771	Maternal Asthma
551	Not Annotated	228.1594	4.72	0.441138275	Medians at zero
580	Not Annotated	228.1959	4.99	0.014078917	No Maternal Asthma
714	Spectral Match to 9(10)-EpOME from NIST14	279.2322	6.51	0.63021781	No Maternal Asthma
879	10E,12Z-octadecadienoic acid	281.2487	8.00	0.596189921	Maternal Asthma
985	Linoleic acid methyl ester	263.2379	8.01	0.500581352	Maternal Asthma
1008	13-Keto-9Z,11E-octadecadienoic acid	295.2270	6.72	0.289838718	No Maternal Asthma
1012	Not Annotated	283.2653	8.44	0.24792309	Maternal Asthma
1082	Spectral Match to 9(10)-EpOME from NIST14	279.2328	7.62	0.24792309	Maternal Asthma
1084	Oleic acid methyl ester	265.2533	8.44	0.101876059	Maternal Asthma
1102	cis-Vaccenic acid	565.5209	8.44	0.083264517	Maternal Asthma
1120	Not Annotated	255.2328	7.80	0.026885512	Maternal Asthma
1126	cis-5,8,11,14-Eicosatetraenoic acid	305.2480	7.94	0.289838718	Maternal Asthma
1130	Spectral Match to Monoolein from NIST14	357.3009	8.01	0.034153957	Maternal Asthma
1150	Not Annotated	239.2377	8.32	0.289838718	No Maternal Asthma
1159	Not Annotated	247.2425	8.44	0.083264517	Maternal Asthma
1172	Conjugated linoleic acid (9E,11E)	263.2379	7.50	0.083264517	Maternal Asthma
1173	1-Linoleoylglycerol	355.2847	7.58	0.500581352	No Maternal Asthma
1194	cis-8,11,14-Eicosatrienoic acid	307.2635	8.21	0.067508162	Maternal Asthma
1216	Not Annotated	265.2533	7.93	0.026885512	Maternal Asthma
1543	trans-13-Octadecenoic acid	283.2648	7.12	0.277428975	No Maternal Asthma
1577	Not Annotated	300.1807	4.82	0.101876059	Maternal Asthma
1715	Linoelaidic acid	245.2271	8.00	0.067508162	Maternal Asthma
1796	Not Annotated	269.2482	6.80	0.177931725	Maternal Asthma
1853	Spectral Match to Monopalmitolein (9c) from NIST14	329.2704	7.34	0.005262242	Maternal Asthma
1943	10E,12Z-octadecadienoic acid	281.2480	6.76	0.386185032	No Maternal Asthma
1955	Linoleic acid methyl ester	263.2376	6.78	0.441418327	No Maternal Asthma
1990	Spectral Match to Cholestan-3-one, (5.alpha.)- from NIST14	369.3509	10.43	0.736121263	No Maternal Asthma
2171	Oleic acid methyl ester	265.2541	7.12	0.111910889	No Maternal Asthma
2380	10E,12Z-octadecadienoic acid	281.2493	7.35	0.018326257	No Maternal Asthma
2385	Not Annotated	297.2804	9.37	0.003870572	Maternal Asthma
2386	Not Annotated	295.2635	8.94	0.005262242	Maternal Asthma
2397	Not Annotated	255.2323	6.42	0.248213079	No Maternal Asthma
2435	9-Oxo-10(E),12(E)-octadecadienoic acid	277.2167	6.25	0.92334184	No Maternal Asthma
2498	Not Annotated	333.2792	8.40	0.101876059	No Maternal Asthma
2537	Not Annotated	295.2271	5.84	0.12365771	No Maternal Asthma
2560	Not Annotated	329.2487	7.85	0.386476231	Maternal Asthma
2584	Spectral Match to 9-Oxo-10E,12Z-octadecadienoic acid from NIST14	277.2169	5.84	0.386476231	No Maternal Asthma
2635	cis-12-Octadecenoic acid methyl ester	247.2439	7.11	0.360055018	No Maternal Asthma
2691	10E,12Z-octadecadienoic acid	281.2480	6.13	0.026885512	No Maternal Asthma
2706	Not Annotated	295.2273	4.67	0.083264517	Maternal Asthma
2724	Not Annotated	395.3707	7.75	0.386185032	No Maternal Asthma
2871	Not Annotated	277.2166	4.67	0.289838718	No Maternal Asthma
2882	Not Annotated	187.0961	2.62	0.177931725	No Maternal Asthma
2898	Not Annotated	299.2589	6.77	0.630427502	No Maternal Asthma
2915	Not Annotated	195.1371	4.68	0.067508162	No Maternal Asthma
2972	Not Annotated	237.2218	6.38	0.210961629	No Maternal Asthma
3041	Spectral Match to 1-Stearoyl-2-hydroxy-sn-glycero-3-phosphocholine from NIST14	311.2969	8.36	0.885164015	No Maternal Asthma
3167	Not Annotated	395.3704	9.17	0.736121263	Medians at zero
3371	Not Annotated	423.3639	9.14	0.84738966	Medians at zero
3455	Linoleic acid methyl ester	245.2274	6.78	0.92334184	No Maternal Asthma
3588	Not Annotated	601.5547	7.10	1	Medians at zero
3674	Cholic acid	373.2750	5.18	0.441418327	No Maternal Asthma
3708	Cholic acid	355.2640	5.19	0.500581352	No Maternal Asthma
3738	4.beta.-Hydroxycholesterol 4-acetate	367.3380	8.67	0.92334184	Medians at zero
3744	Not Annotated	309.2810	8.00	0.700311373	No Maternal Asthma
3749	Not Annotated	583.5318	10.38	0.083264517	Maternal Asthma
3811	Not Annotated	297.2813	8.07	0.386476231	Maternal Asthma
3896	Not Annotated	329.3081	8.35	0.563702862	Medians at zero
4057	Not Annotated	309.2800	9.23	0.0160797	Maternal Asthma
4141	Not Annotated	601.5430	7.12	0.335923813	Maternal Asthma
4353	Not Annotated	483.4420	10.46	0.004505985	Maternal Asthma
4526	Not Annotated	381.3526	8.90	0.92334184	Medians at zero
4623	5.alpha.-Pregnane-3.alpha.,20.alpha.-diol	285.2595	5.02	0.700311373	Medians at zero
4626	Not Annotated	291.1954	5.82	0.148914673	No Maternal Asthma
4635	"(4R)-4-((3R,5R,6S,9S,10R,13R,14S,17R)-3,6-dihydroxy-10,13-dimethylhexadecahyd	357.2799	5.87	0.630427502	No Maternal Asthma
4639	.beta.-Hyodeoxycholic acid	375.2895	5.88	0.809779771	Medians at zero
4670	Not Annotated	297.2427	5.47	0.289838718	No Maternal Asthma
4733	Not Annotated	379.2855	7.50	0.012354585	Maternal Asthma
4748	Not Annotated	601.3381	7.08	0.84738966	Medians at zero
5049	Not Annotated	353.2694	6.15	0.563702862	Medians at zero
5138	Not Annotated	383.3325	5.00	0.083264517	No Maternal Asthma
5144	Conjugated linoleic Acid (10E,12Z)	281.2482	7.37	0.067508162	Maternal Asthma
5281	Spectral Match to 9(10)-EpOME from NIST14	279.2324	7.62	0.700311373	Maternal Asthma
5282	Spectral Match to 9-OxoOTrE from NIST14	275.2018	6.35	0.12365771	No Maternal Asthma
5376	Not Annotated	317.2685	5.61	0.177931725	No Maternal Asthma
5403	Not Annotated	601.2194	7.15	0.92329497	Medians at zero
5453	Not Annotated	293.2119	4.36	0.885164015	Medians at zero
5499	Not Annotated	295.2657	7.70	0.84738966	No Maternal Asthma

Supplementary Table 4: Milk feed composition of the two groups at each timepoints. P-value for timepoint 2 was 1.0 and timepoint 3 was 0.31. Statistical analysis was performed using a Chi-square test.

Milk Feed Composition		Maternal Asthma, n = 9	Control, n = 9
Timepoint 2	Maternal milk	1	2
	Fortified maternal milk	7	6
	Fortified donor milk	1	1
	Fortified mixed maternal and donor milk	0	0
	Preterm formula	0	0
	Unknown	0	0
Timepoint 3	Maternal milk	0	1
	Fortified maternal milk	4	3
	Fortified donor milk	1	1
	Fortified mixed maternal and donor milk	1	0
	Preterm formula	1	4
	Unknown	2	0

Supplementary Table 5: Metabolomics features identified as important for sample classification using the supervised learning method, QIIME 2 sample classifier. Metabolites are listed in order of importance score, identified by their GNPS feature ID. Annotations provided if matched using the GNPS library spectra search. Metabolites found on the linoleic acid network in Figure 5 are highlighted in yellow.

feature_ID	importance score	Annotation		feature_ID	importance score	Annotation
3416	0.01333222			2961	0.002699844	
2587	0.012024048			3235	0.002699844	
5521	0.008016032			3445	0.002699844	
5359	0.007014028			4602	0.002699844	
1458	0.006012024			700	0.002691096	
2182	0.006012024			3444	0.002691096	
3941	0.006012024			4192	0.002691096	
5494	0.006012024			4257	0.002691096	
3391	0.005316188			5013	0.002691096	
4370	0.005316188			5069	0.002691096	
4146	0.004703852			5071	0.002691096	
2258	0.004695104			3352	0.002661573	
608	0.004008016			3490	0.002661573	
2410	0.004008016			4961	0.002661573	
3328	0.004008016			2481	0.002384929	NCGC00186665-03!2,3-dihydroxypropyl hexadecanoate
4254	0.004008016			2854	0.002384929	
4301	0.004008016			2985	0.002384929	
4529	0.004008016			3889	0.002384929	
4732	0.004008016			4580	0.002376181	
4842	0.004008016			1525	0.002348447	
5223	0.004008016			2691	0.002318924	10E,12Z-octadecadienoic acid
768	0.003701848			4057	0.002310176	
1537	0.003701848			911	0.002067793	
4317	0.003701848			4466	0.002042279	
4679	0.0036931			2246	0.002004008	
5533	0.0036931			2364	0.002004008	
4267	0.00339568			2419	0.002004008	
2911	0.003350451			2672	0.002004008	
3549	0.003320928			2811	0.002004008	
2380	0.00331218	10E,12Z-octadecadienoic acid		2815	0.002004008	
1055	0.003006012			2820	0.002004008	
2688	0.003006012			2946	0.002004008	
2696	0.003006012			2981	0.002004008	
3305	0.003006012			2986	0.002004008	
3319	0.003006012			3112	0.002004008	
3437	0.003006012			3397	0.002004008	
3541	0.003006012			3486	0.002004008	
3570	0.003006012			4013	0.002004008	
4690	0.003006012			4531	0.002004008	
4805	0.003006012			4532	0.002004008	
4925	0.003006012			4740	0.002004008	
5067	0.003006012			4825	0.002004008	
5156	0.003006012			4853	0.002004008	
5292	0.003006012			4880	0.002004008	
5396	0.003006012			4892	0.002004008	
5399	0.003006012			5032	0.002004008	
5536	0.003006012			5072	0.002004008	
4077	0.002967741	Linoleic acid ethyl ester		5100	0.002004008	
4969	0.002699844			5153	0.002004008	
5469	0.002699844			5176	0.002004008	
